# P07-06 Physical activity promotion in cancer patient: opinions and practices of health professionals

**DOI:** 10.1093/eurpub/ckac095.106

**Published:** 2022-08-29

**Authors:** Yoric Petitfrère, Jérôme Rompen, Marc Cloes, Alexandre Mouton

**Affiliations:** Motor Sciences, University of Liège, Liège, Belgium

**Keywords:** cancer, health professionals, health promotion

## Abstract

**Background:**

Literature suggests that health professionals (HPs) can play an important role in promoting physical activity (PA) in cancer patients (Jones et al., 2004). This study had three purposes: (1) analyzing HPs' knowledge and opinion about PA for cancer patients; (2) examining practices, barriers and roles that they identify when considering PA promotion in their patients; (3) determining factors that may enhance practices in this field.

**Methods:**

This study was designed in two phases and took place in Wallonia, Belgium. The first phase was qualitative: 12 HPs were interviewed about their experiences with PA in cancer patients. Then, in the second (and quantitative) phase, an online survey was created based upon the results of the first phase and upon the relevant literature. We sent this second-phase survey to professional organizations, hospitals and medical health centers and received 68 responses.

**Results:**

In both phases of the study, HPs seemed to be aware of the benefits of PA for cancer patients and considered that they have to play a role in promoting PA in that specific population. However, only 25% (n = 17) of HPs were familiar with the official PA guidelines. According to our results, HPs discuss the topic of PA with more than 7 out of 10 patients (7.11 ± 2.61). Lack of time and lack of knowledge about PA were identified as barriers to PA promotion. Finally, other relationships that may help to explain the role of HPs in PA promotion were uncovered: clinicians who used more information (e.g. PA benefits) (p = >0.001), exercised referrals (p = 0.012), and prescribed PA (p = 0.007) had higher intervention rates.

**Conclusions:**

Our findings suggest that improving HPs' knowledge about PA guidelines and how it can be proposed to cancer patients could help them to promote PA and to encourage their patients to consult a PA specialist, and eventually adopt a physically active lifestyle.

